# Trends and cross-country inequalities in dengue, 1990–2021

**DOI:** 10.1371/journal.pone.0316694

**Published:** 2025-06-20

**Authors:** Mingzhu Zhou, Yong He, Liangmiao Wu, Kaiyuan Weng

**Affiliations:** 1 School of Pharmaceutical Business, Guangdong Pharmaceutical University, Guangzhou, Guangdong Province, China; 2 Jinan University First Affiliated Hospital, Guangzhou, Guangdong Province, China; Universitas Muhammadiyah Aceh, INDONESIA

## Abstract

**Background:**

The existing body of literature is deficient in the most recent data regarding the global perspective of dengue fever and its associated health inequities.Our aim is to assess the global burden of dengue fever and its health inequities from 1990 to 2021.

**Methods:**

The Global Burden of Disease (GBD) database, organized by global health research institutions, is a comprehensive database and the largest-scale and most detailed scientific research to date. We utilized the GBD database for epidemiological trends, demographic analysis, epidemiological decomposition, cross-national inequality and predictive modeling for the global dengue burden up to 2051 were also performed.

**Results:**

Globally, dengue fever incidence, prevalence, Disability-Adjusted Life-Years (DALYs), and mortality have risen with marked international disparities. From 1990 to 2021, Age-Standardized Rates (ASRs) for incidence and prevalence rose by 1.83% (95% CI: 1.58%–2.08%), and for DALYs and mortality by 1.33% (95% CI: 1.10%–1.57%) and 1.70% (95% CI: 1.45%–1.94%), respectively. Age-Period-Cohort (APC) model analysis showed a positive link between dengue fever incidence and age, with mortality spiking in those over 80. DALYs burden decomposition highlighted population growth as the key driver of global burden, yet impacts differed across Socio-Demographic Index (SDI) quintiles. Dengue fever burden inequalities related to SDI have remained, with benefits shifting from poor to rich populations. Bayesian Age-Period-Cohort (BAPC) model projections indicate stable incidence and prevalence ASRs, but declining DALYs and mortality ASRs, especially for females.

**Conclusion:**

This study elucidates the changes in the burden of dengue fever against the backdrop of a burgeoning global population, severe aging, and pronounced health inequities across nations, quantifying these alterations and forecasting the trends in the disease burden over the next three decades. Concurrently, the research proposes effective measures for various countries and regions to mitigate health inequities.

## Introduction

Dengue fever, an acute vector-borne infectious disease caused by the Dengue virus, poses a significant challenge to public health on a global scale [1]. This viral disease is primarily transmitted by the *Aedes aegypti* and *Aedes albopictus* mosquitoes, with approximately 390 million people infected annually, out of which 96 million cases exhibit clinical symptoms [2]. As global climate change and urbanization accelerate, the endemic regions of dengue fever are expanding, and the disease burden is increasing, particularly in tropical and subtropical areas [3].

From a virological perspective, the Dengue virus has four distinct serotypes, namely DENV-1, DENV-2, DENV-3, and DENV-4 [4–6], and exhibits a high mutation rate, with each genotype potentially forming its own quasi-special group [7]. Vaccine developers must address the challenge of ensuring that their candidate vaccines induce a strong neutralizing immune response against all serotypes without causing antibody-dependent enhancement (ADE) [8]. Regrettably, despite the availability of vaccines against Dengue fever [9], the implementation of vaccination in highly endemic regions must also consider the sero prevalence in the target population [10]. Most importantly, without early detection and proper medical care, DENV can lead to fatal outcomes such as Dengue fever (DF), Dengue hemorrhagic fever (DHF), and Dengue shock syndrome (DSS) [11], with the mortality rate for severe Dengue infections potentially exceeding 20% [12].

Due to the significant negative impact of dengue fever, existing studies have attempted to address the issue by identifying the determinants of dengue incidence [13,14], predicting its incidence [15,16], and estimating the resulting economic burden [17,18]. However, they have overlooked the varying impacts of different public health policies across countries on the incidence, prevalence, Disability-Adjusted Life Years (DALYs), and even mortality rates of dengue fever among different age groups.

Understanding the geographical distribution and disease burden of dengue fever, as well as the public health policies of different countries, is crucial for determining how to optimally allocate limited resources available for dengue control and for assessing the international impact of such activities [19]. Previous research has indicated that the unequal distribution of disease burden places greater health and economic pressures on resource-poor areas and vulnerable populations. This inequality is not only evident between different countries and regions but also among different socioeconomic groups.

This study will employ a multidisciplinary approach, integrating epidemiological and public health policy research, to conduct an in-depth analysis of the trends and inequalities in the global disease burden of dengue fever. By collecting and analyzing global dengue case data, the aim is to reveal the characteristics of the global and regional distribution of the dengue disease burden, identify key factors contributing to disparities in disease burden, and explore potential strategies to reduce health inequalities. Through this research, we anticipate providing valuable information and recommendations to global health policymakers, in order to achieve more equitable and effective global health governance.

## Methods

### Data source

The GBD 2021 utilized the most recent epidemiological data and improved standardization methods to comprehensively assess health losses from 371 diseases, injuries, and risk factors across 204 countries and territories, stratified by age and sex [20,21]. GBD 2021 integrated various data sources, each with a unique identifier, listed in the Global Health Data Exchange (GHDx). The collected data were modeled using Spatio-Temporal Gaussian Process Regression (ST-GPR), allowing for the smoothing of age, time, and location in areas lacking complete datasets [10].

For epidemiological data with known biases, the Meta-regression-Bayesian (MR-BRT) model was employed to adjust for biases resulting from research methodologies. Incidence and prevalence were modeled using DisMod-MR 2.1 (Disease Modeling Meta-Regression; version 2.1), while DALYs were estimated by location, age, sex, and year, and mortality was modeled using the Cause of Death Ensemble model (CODEm). In this study, estimates and their 95% uncertainty intervals (UI) were derived from the GBD 2021 database [20]. We extracted data on the number of reported dengue cases and the age-standardized incidence, prevalence, DALYs, and mortality rates for males, females, and both sexes combined at the global, regional, and national levels. All rates are reported per 100,000 people. Additionally, the attributable burden estimates were stratified by Socio-demographic Index (SDI) quintiles, representing a composite measure of income, education, and fertility conditions, quantifying the socio-demographic development level of a country or region, including five SDI quintiles (i.e., low, low-middle, middle, high-middle, high), representing five levels of development, and presented in counts, age-standardized rates, and rankings.

### Trend analysis

The trend in Age-Standardized Rates (ASR) measured by the Annual Percentage Change (EAPC) is a more reliable indicator for monitoring changes in disease patterns [22]. A linear regression model was constructed using R version 4.3.2, specified as (y = α + *β* x + ∊), where (y = ln(ASR)), (x) represents the calendar year, and (∊) is the error term, with (*β*) indicating the positive or negative trend in ASR. The EAPC was then calculated as ((exp(*β*) – 1) × 100%), and its 95% confidence interval (CI) was derived from the model [23]. If the EAPC estimate and the lower limit of its 95% CI are both >0, the ASR is considered to be increasing. Conversely, if the EAPC estimate and the upper limit of its 95% CI are both <0, the ASR is decreasing. Otherwise, the ASR is considered stable.

After preprocessing the relevant data using R code, we employed the Age-Period-Cohort Analysis Web Tool to analyze the temporal trends of a variable. This tool integrates three temporal dimensions—age, period, and cohort—to estimate their effects on incidence and mortality rates. An age-period-cohort (APC) model studies the changes in a variable over time by simultaneously including three temporal dimensions to estimate the effects of age, period, and birth cohort on incidence and mortality rates. In this model, the age effect represents changes in the variable throughout an individual’s life, while the period effect represents the impact of environmental factors affecting the entire population. Additionally, the birth cohort effect refers to changes in the variable due to similar life events experienced by those born in the same year [24,25]. However, the inevitable identification issues arising from multicollinearity between ages, periods, and birth cohorts lead to challenges in estimating the effects of each. To address this, an Intrinsic Estimator (IE) method for APC models is required [26,27], which uses principal component regression analysis to explain the variation effects on the three temporal trends and provides relatively efficient estimates. The IE method’s APC model is based on the Poisson distribution. Given that the latest available data in the GBD database spans from 1990 to 2021 (a total of 32 years), we excluded data from 1990 and 1991 to facilitate modeling and subsequent analyses. We then categorized the data at 5-year intervals for age groups (e.g., under 5 years, 5–10 years,..., 90–95 years, and over 95 years). Correspondingly, we created 5-year period groups (e.g., 1992–1996, 1997–2001,..., 2012–2016, 2017–2021) and derived the associated birth cohorts (e.g., 1892–1896, 1897–1901,..., 2017–2021) and by comparing incidence and mortality in the same age group across different periods to estimate the net age, period, and birth cohort effects on dengue fever incidence and mortality rates. The IE method’s APC model provides the effects of age, period, and birth cohort. These coefficients are then transformed into exponent values to determine the relative risks (RRs) of incidence and mortality at specific ages, periods, or birth cohorts compared to the average combined level across all ages, periods, or birth cohorts [28].

### Decomposition analysis

To elucidate the contributing factors driving the changes in dengue fever DALYs from 1990 to 2021, we conducted a decomposition analysis stratified by sex, at the global and SDI quintile levels, accounting for demographic shifts, age structure, and epidemiological changes. Initially, DALYs for dengue fever were categorized into three subgroups based on gender: male, female, and all-gender. Subsequently, the DALYs rate, defined here as epidemiological change, was calculated as described by Xie et al. [29]: the number of DALYs at location, age, and year (DALYs_ay, py, ey_) can be computed as follows: DALYs_ay, py, ey_ = ∑^n^_i=1_(a_i,y_ * p_y_ * e_i,y_), where DALYs a_y_, p_y_, e_y_ represents the DALYs influenced by age structure, population, and DALYs rate for year y; a_i,y_ is the population fraction for age group i of n groups in year y; p_y_ is the total population for year y; and e_i,y_ is the DALYs rate for age group i in year y. For instance, the impact of age structure is calculated as: [(DALY_a2021, p1990, e1990_ + DALY_a2021 p2021, e2021_)/3 + (DALY_a2021, p1990, e2021_ + DALY_a2021, p2021, e1990_)/6] – [(DALY_a1990, p2021, e2021_ + DALY_a1990, p1990, e1990_)/3 + (DALY_a1990, p2021, e1990 _+ DALY_a1990, p1990, e2021_)/6]. The determination of their impact on population and epidemiological changes is identical.

### Cross-country inequalities analysis

Monitoring health inequalities can provide a foundation for evidence-based health planning, further improving policies, programs, and practices to reduce disparities in health distribution. In this study, we employed two standard indicators of absolute and relative gradients of inequality, namely the Slope Index of Inequality (SII) and the Concentration Index, to measure the inequality of dengue fever burden among countries and analyze the trends in global health inequalities from 1990 to 2021 [30,31]. The Slope Index of Inequality (SII) was calculated through regression analysis to link the DALY rates of countries in 1990 and 2021 with their relative positions on a scale of socio-demographic development. The relative position was determined by the midpoint of the cumulative population range ordered by the SDI. The Concentration Index was calculated by numerical integration of the area under the Lorenz concentration curve, fitting the cumulative fraction of DALYs and the cumulative relative distribution ordered by SDI. The Concentration Index is derived from the concentration curve, which plots the cumulative percentage of socioeconomic status on the x-axis and the cumulative percentage of health variables (e.g., prevalence, mortality) on the y-axis. When health distribution is perfectly equal, the concentration curve coincides with the 45-degree diagonal line. If poor health is concentrated among populations with lower socioeconomic status, the curve lies above the diagonal, yielding a negative Concentration Index value. Conversely, if poor health is concentrated among populations with higher socioeconomic status, the curve lies below the diagonal, resulting in a positive Concentration Index value [32].

### Predictive analysis

The aforementioned analyses focused on the burden of dengue fever over the past decades. To establish better public health policies and allocate health resources rationally, this study further predicted the burden of dengue fever over the next three decades. The Integrated Nested Laplace Approximation (INLA) with the Bayesian Age-Period-Cohort (BAPC) model offers better coverage and precision than the APC model. Therefore, this study utilized INLA with the BAPC model to approximate the marginal posterior distribution, forecasting the different burdens of dengue fever for males and females globally up to 2051 [33,34], avoiding several mixing and convergence issues associated with the Markov Chain Monte Carlo sampling techniques traditionally used for Bayesian methods [33].

All statistical analyses in this study were conducted using R version 4.3.2.

## Results

### Overall trends in dengue burden using broad estimation method and descriptive analysis

**Fig 1:**
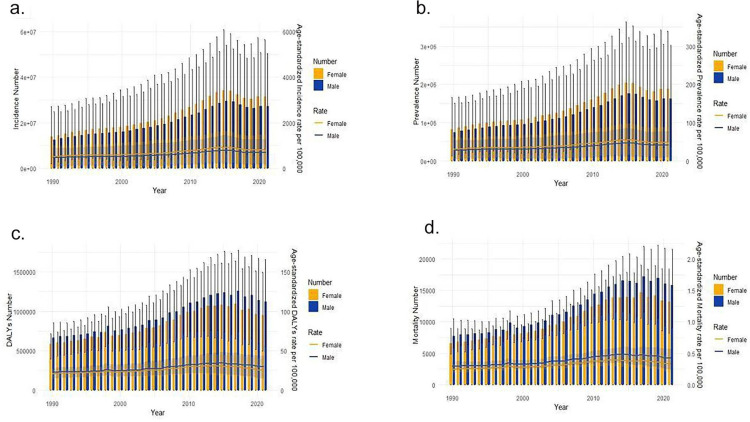
1990-2021 Dengue trend. The number and ASR of incidence (a), prevalence (b) DALYs(c), and mortality(d) in 1990–2021 Dengue Trend. DALYs, disability-adjusted life-years.

**Fig 2:**
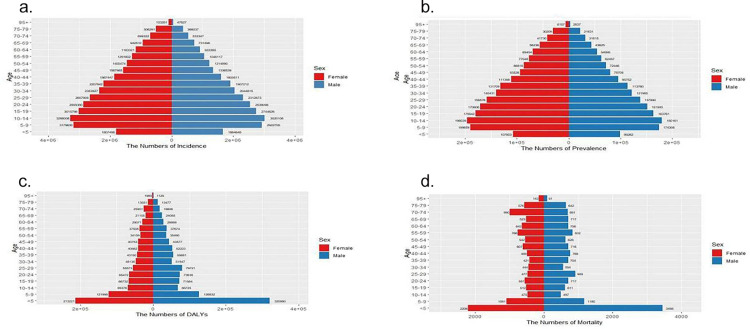
2021 Dengue’s gender-stacked chart. The number of incidence(a), prevalence (b), DALYs (c) and deaths (d) of Dengue in Males and Females in 2021. DALYs, disability-adjusted life-years.

Globally, from 1990 to 2021, the number of dengue fever cases, along with the Annual Incidence Rate (ASIR), Annual Prevalence Rate (ASPR), and DALYs, have all shown an overall increasing trend. Between 1990 and 2021, the ASIR and ASPR for dengue fever both rose by an average of 1.83% (95% CI: 1.58%−2.08%); the ASR for DALYs and mortality rates increased by 1.33% (95% CI: 1.10%−1.57%) and 1.70% (95% CI: 1.45%−1.94%), respectively (S1–S4 Tables). Dual-axis charts reveal that the highest number of dengue fever cases occurred in 2015, and the highest number of dengue-related deaths in 2017 within the scope of this study. The overall development trends for both are similar, yet there is a noticeable lag for the peak in mortality compared to the incidence. Notably, in terms of the impact of DALYs and deaths due to dengue fever, the values for males significantly exceed those for females, while the overall gender trend for incidence and prevalence is inverse (Fig 1). This disparity is more pronounced in the 2021 gender-stacked chart (Fig 2).

At the regional level, the EAPC in the ASIR and ASPR are nearly congruent, with the high-income North America region showing the most significant increasing trend, while the Eastern Sub-Saharan Africa has the fastest decline. In the ASR for DALYs, the EAPC in the high-income North America region and Australia remains significantly elevated, but in the downward trend, Western Europe has taken the lead in place of Eastern Sub-Saharan Africa. Regarding the EAPC for the Age-Standardized Mortality Rate (ASMR), Tropical Latin America has the highest increasing trend, while Southern Latin America has the fastest decline (S1–S4 Tables).

**Fig 3:**
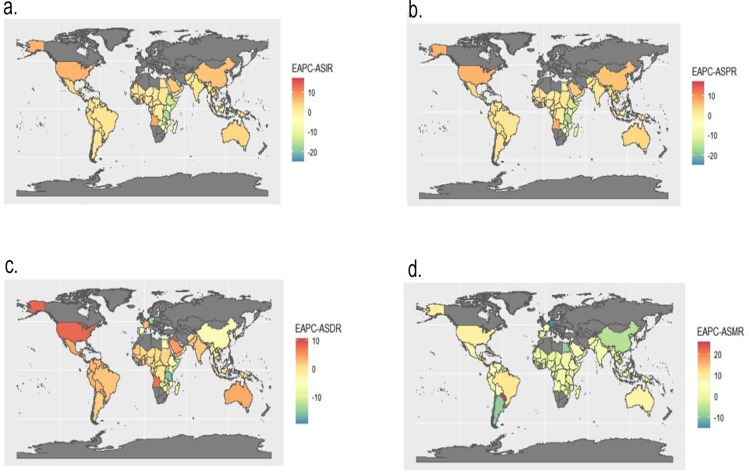
The EAPC trends in ASR for incidence, prevalence, DALYs and mortality. (a) The EAPC Trends in ASIR of Dengue from 1990 to 2021; (b) The EAPC Trends in ASPR of Dengue from 1990 to 2021; (c) The EAPC Trends in ASDR of Dengue from 1990 to 2021; (d) The EAPC Trends in ASMR of Dengue from 1990 to 2021. ASIR, age-standardized incidence rate; ASPR, age-standardized prevalence rate; ASDR, age-standardized disability-adjusted life-years rate; ASMR, age-standardized mortality rate; EAPC, estimated annual percentage change.

At the national level, the overall trends in disease burden vary. Among various countries and regions (Fig 3), the EAPC in the ASR for both incidence and prevalence was highest in Tonga and lowest in South Sudan. However, in the EAPC for the age-standardized Disability-Adjusted Life Years rate (ASDR), Equatorial Guinea exhibited the highest increasing trend, while South Sudan had the most significant decreasing trend. Surprisingly, in the EAPC for the ASMR, Paraguay had the highest increasing trend, and Kuwait was at the forefront of the decreasing trend.

Additionally, when examining the range of SDI quintiles, the EAPC in the ASIR and ASPR for regions in the high-middle SDI quintile showed the most significant increasing trend among all SDI ranges; conversely, the EAPC for ASIR and ASPR in the low SDI quintile exhibited the fastest decline, with both trends being nearly congruent. Regarding the EAPC changes in the ASDR and ASMR due to dengue fever, regions with high SDI experienced the fastest rate of increase. However, in the EAPC for ASDR, regions with low SDI also showed an increasing trend, albeit at the slowest rate. The EAPC for ASMR showed the least change in regions with high-middle SDI (S1–S4 Tables).

### Age-period-cohort analysis on dengue incidence and prevalence

**Fig 4:**
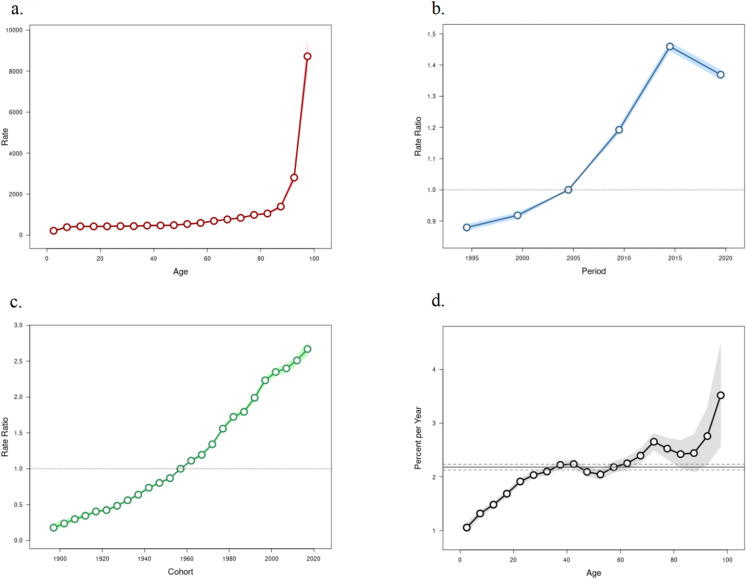
Dengue’s incidence age-period-cohort model (1). Longtidunal age curve (a); period relative rate (b); cohort relative rate (c); local drifts with net drift (d); of incidence. RR, relative risk.

**Fig 5:**
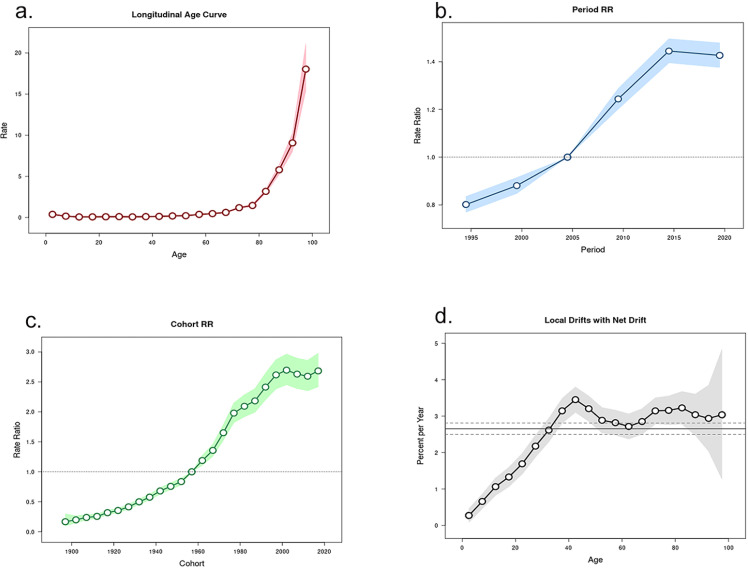
Dengue’s mortality age-period-cohort model (2). Longtidunal age curve (a); period relative rate (b); cohort relative rate (c); local drifts with net drift (d); of mortality. RR, relative risk.

The results of age-period-cohort analysis for the incidence and mortality of dengue fever are presented in Figs 4 and 5.

After controlling for period and birth cohort effects, the age effect was shown to significantly influence the incidence and mortality of dengue fever, with both being positively correlated with age. Among those under 80 years old, the impact of age on the prevalence and mortality of dengue fever was relatively flat, while in individuals over 80 years old, the incidence and mortality rates of dengue fever increased linearly.

After controlling for age and birth cohort effects, the period effect had a significant impact on the incidence and mortality of dengue fever. The model used the incidence rate of dengue fever in 2005 as the baseline level, showing a long-term increasing trend from 2005 to 2015, with a relative risk (RR) increase of approximately 1.5 times compared to 1990, and serving as an inflection point for the subsequent decline in RR values. The mortality rate of dengue fever consistently showed an increasing trend from 1990 to 2015, and it was not until after 2015 that a decline in mortality rates began to emerge.

After controlling for the effects of age and period, the birth cohort effect significantly influences the risk of incidence and mortality of dengue fever. The birth cohort effect suggests that compared to earlier cohorts, individuals born between 2015 and 2019 have the highest risk of developing the disease. Moreover, as time progresses, the relationship between the RR and time approximates a directly proportional function trend.

It is noteworthy to compare the incidence of dengue fever across different time periods within the same age groups. The results indicate a sustained increase in the incidence of dengue fever among individuals aged 0–40, while those aged 40–45 continue to show an upward trend, albeit at a stable rate. A turning point is observed in the upward trend among individuals over the age of 70, with a subsequent sharp increase in the incidence of dengue fever in those over 90. Mortality rates, compared to the same point in the previous year, exhibit an almost directly proportional increasing trend in individuals aged 0–45. For those aged 45–60, the rate of increase slows down relative to the baseline level. However, for individuals over 60, mortality rates continue to fluctuate upwards, with an overall trend of continuous increase (S5 and S6 Tables).

### Decomposition analysis on dengue DALYs

**Fig 6:**
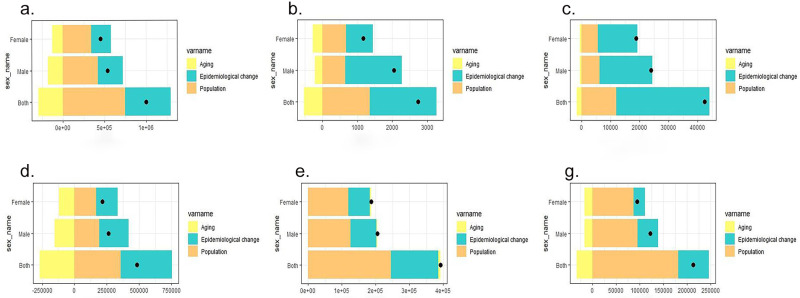
Decomposition analysis on dengue DALYs. Changes in DALYs of dengue according to aging, population growth and epidemiological change from 1990 to 2021 at global level by SDI quintile and by subgroups of sexes. The black dot denotes the overall value of the changing resulting from all three components. For each component, the magnitude of a positive value suggests a corresponding increase in dengue DALYs attributed to the component; the magnitude of a negative value suggests a corresponding decrease in dengue DALYs attributed to the component. DALYs, disability-adjusted life-years; SDI, socio-demographic index.

Over the past 32 years, there has been a sharp global increase in DALYs, with varying impacts of aging, population growth, and epidemiological changes across different SDI quintiles. Notably, the highest increase was observed in the middle SDI quintile. As of 2021, global aging contributed a negative growth of 29.55%, while population and epidemiological changes accounted for 74.45% and 54.80% of the global increase, respectively. The most significant contributions of aging, population growth, and epidemiological changes were noted in the middle SDI quintile with a negative growth of 58.33%, a growth of 92.41% in the low SDI quintile, and a growth of 85.08% in the middle SDI quintile, respectively (S7 Table). When stratified by gender, the impacts of aging, demography, and epidemiology on DALYs varied across subgroups, with the burden being higher in males across all subgroups. The influence of population and epidemiology on DALYs was most pronounced in all SDI quintiles across all subgroups. Interestingly, the negative impact of aging was most pronounced in the middle SDI quintile, population growth had a significant impact on low-middle SDI regions, and the changes in DALYs due to dengue fever did not follow a simple ranking according to the definition of the quintiles from high to low (Fig 6).

### Cross-country inequality analysis

**Fig 7:**
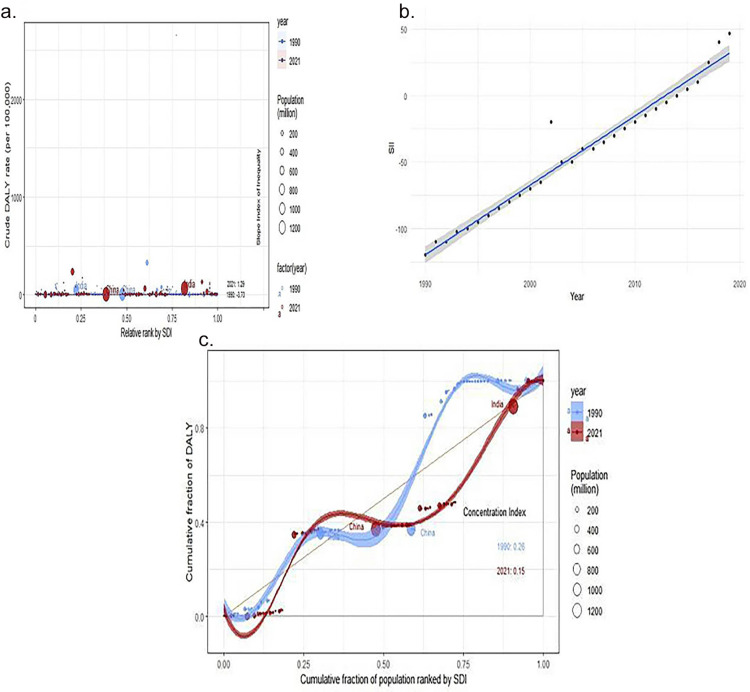
Analysis of cross-country inequality health. SDI-related health inequality regression for the DALYs of Dengue worldwide, 1990 and 2021 (a); Trends in the SII for Dengue from 1990 to 2021 (b); concentration for the DALYs of Dengue worldwide, 1990 and 2021 (c). DALYs, disability-adjusted life-years; SDI, socio-demographic index, SII, Spatiotemporal Index of Infection.

Absolute inequalities in the burden of dengue fever in relation to the SDI were observed, with the slope index of inequality for 2021 being 1.29 (95% CI: −0.76 to 3.34), compared to −3.70 (95% CI: −4.94 to −2.46) in 1990. The impact of the SDI on the disease burden of dengue fever has diminished, with greater equality in 2021. Over time, from 1990 to 2014, the inequality in the burden of dengue fever, as measured by the number of reported cases worldwide, gradually decreased and even tended toward equality. However, during the subsequent period from 2015 to 2021, inequality intensified again, and the beneficiary groups shifted from subgroups in poorer groups to those in wealthier groups. Concurrently, the concentration index, which measures relative gradient inequality, was 0.26 (95% CI: 0.21–0.29) in 1990 and, when taken as an absolute value in 2021, was 0.15 (95% CI: 0.13 to 0.17). This indicates an improvement in the relative inequality of the burden of dengue fever, with a clear shift in the advantaged groups, namely from the poor in 1990 to the wealthy in 2021 (Fig 7).

### Predictive analysis on Dengue burden to 2051

**Fig 8:**
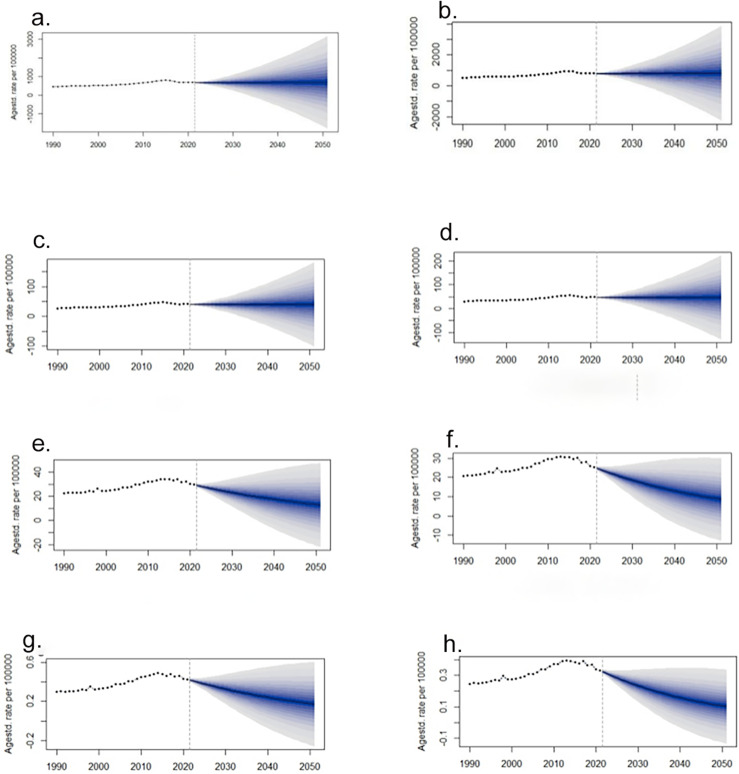
Predictive analysis on dengue burden. (a) The predicated ASIR for males from to 2051; (b) the predicated ASIR for females from to 2051; (c) the predicated ASPR for males from to 2051; (d) the predicated ASPR for females from to 2051; (e) the predicated ASDR for males from to 2051; (f) the predicated ASDR for females from to 2051; (g) the predicated ASMR for males from to 2051; (h) the predicated ASMR for females from to 2051; of dengue globally. ASIR, age-standardized incidence rate; ASPR, age-standardized prevalence rate; ASDR, age-standardized disability-adjusted life-years rate; ASMR, age-standardized mortality rate; DALYs, disability-adjusted life-years.

The ASR for incidence, prevalence, DALYs, and mortality of dengue fever projected to 2051 are depicted in Fig 8. Globally, the ASR for incidence and prevalence is anticipated to stabilize, while the ASR for DALYs and mortality is projected to continue declining through 2051, with a more pronounced decline observed in females compared to males. Detailed values can be found in S8 Table.

## Discussion

Through the comparative analysis presented, this study provides a detailed examination of the global, regional, and national burden of dengue fever from 1990 to 2021, based on trends, decomposition, health inequalities, and predictive analysis. Although there are differences in incidence, prevalence, DALYs, and mortality rates across countries, the global burden of dengue fever has generally shown an increasing trend from 1990 to 2021, with a shift in the beneficiary groups of health inequality during this period.

In terms of SDI quintiles, regions with middle SDI exhibited the highest incidence and mortality rates. Other regions showed a trend towards convergence towards the middle values. Although low-SDI regions still bear a heavy burden, a declining trend has been observed. Conversely, high-SDI regions have shown an increasing trend, with the gap between the two narrowing. The burden of dengue fever is no longer solely a concern for developing countries; developed countries such as the United States and Australia also face significant disease burdens. The rapid increase in dengue incidence in middle and high-middle SDI regions is consistent with previous studies [35]. However, we were surprised to find that the most significant upward trends in DALYs and mortality rates were observed in high-SDI regions. This may be attributed to frequent international travel and trade, which facilitate the introduction of the dengue virus. Despite the relative abundance of medical resources in high-SDI regions, uneven distribution limits timely access to care for some patients.

From a GBD regional perspective, compared to previous research on dengue fever [36,37], firstly, North America has become a hotspot for the spread of dengue fever. However, North America is not a geographically specific region for dengue fever transmission; the extensive spread of dengue fever in this area is often related to population movements, travel, and trade between countries [38,39]. Global climate change is a significant driver of dengue transmission, altering its seasonal patterns and expanding its geographical range [35]. For instance, in temperate regions such as northern Asia and central and northern Europe, increased climatic suitability has heightened the risk of *Aedes aegypti* mosquito incursion and, consequently, dengue transmission [40,41]. In tropical Latin America, dengue remains the leading cause of mortality globally, likely due to the region’s vulnerability to El Niño events, inconsistent government approaches to vaccination, and the lack of community-centered mosquito control measures [42]. In contrast, dengue mortality rates have declined in Western Europe and South America, potentially due to heightened global attention to dengue and the impetus provided by the WHO’s “Non-Communicable Diseases Roadmap” and the “London Declaration” [43,44].

Nationally, it is imperative to devise flexible health policies tailored to the specific circumstances of each country. For instance, Bali, a tourist destination in Indonesia, may not present travelers with a noticeable risk of mosquito bites during their visit, but many often contract dengue fever symptoms after returning from their trip [35]. For Indonesia, effective surveillance and control of the disease among the traveling population is an urgent priority. Concurrently, it is noteworthy that the United States, as a leading economic power, has experienced an annual change in dengue fever incidence that has surpassed that of China. The focus of dengue fever prevention and control in the U.S. appears to be primarily on the 9–45 age group with a history of confirmed dengue infection [45], yet other high-risk age groups susceptible to the disease should be given particular attention through vaccination.

In the existing literature, there is a relative scarcity of studies that conduct a comprehensive analysis of the full age range of incidence and mortality data for dengue fever [46]. This study employs an APC model for analysis and finds that individuals aged 60 and above have become a high-risk group for dengue fever infection and mortality. This phenomenon may be associated with the prevalence of chronic diseases, malnutrition, and decreased immunity among the elderly, all of which can exacerbate the disease burden. Therefore, special attention should be given to this group in disease prevention and control strategies [47, 48, 49]. Additionally, the bilateral chart analysis of this study for the year 2021 reveals that the number of dengue fever deaths among children and infants under the age of 10 remains high. This may be related to complex socio-political factors such as warfare, which could lead to a sharp increase in dengue fever mortality in this age group. Consequently, public health intervention measures should also give special consideration to children in this age range.

As the global population ages and continues to grow, epidemiological shifts occur rapidly, impacting different countries and regions in varying ways based on their economic levels and population dynamics. In regions with higher economic development, better access to public health resources leads to a reduced impact from population-related factors. At this juncture, without external policy interventions that highlight health equity, the disparities in health equity caused by epidemiological changes between impoverished and affluent regions will widen. Particularly for regions with high SDI and middle SDI, the disease burden of dengue fever exacerbated by population aging will further increase.

More importantly, the beneficiary groups of health equity may also shift due to policy differences between regions. Countries with high SDI levels have greater access to health care systems and health care systems perform better, thus potentially incurring a lower burden of disease [50]. According to this study, the health gap between the poorest and richest groups of dengue fever remains large, and the beneficiary group has shifted from poor to affluent groups. To promote health equity, it is imperative to bridge the vaccine divide in the international community. We should intensify vaccine research and development to promote the use of next-generation vaccines, and conduct serological screening prior to vaccination to avoid the risk of ADE [51,52]. Additionally, efforts should be made to enhance vaccine accessibility, particularly in low-income countries, through collaborations between international organizations and governments to reduce costs associated with vaccine supply, cold-chain transportation, and administration. This can be achieved through global cooperation, sharing vaccine development and prevention experiences, especially in resource-limited settings. In the future, as more candidate vaccines enter clinical trial stages, the development of safer and more effective dengue vaccines is anticipated.

Although current predictive models indicate a potential decline in the ASR of DALYs and mortality for dengue fever by 2051, our study’s model provides a range of values that account for the multifactorial influences on dengue incidence and reporting. The potential for numerous factors to exacerbate the situation underscores the uncertainty in projecting the disease burden of dengue fever over the coming three decades. The incidence and reporting of dengue fever is still influenced by multiple factors. During the COVID-19 pandemic, for example, the number of cases reported to WHO decreased during this period [53,54]. As a result, in 2023, another surge in dengue cases was observed worldwide. It is characterized by an increase in number and scale and the simultaneous occurrence of multiple outbreaks that have spread to areas previously unaffected by dengue. In addition, most patients with this disease have primary asymptomatic infection, and most of them do not develop clinical symptoms until the secondary infection. At the same time, dengue reporting is not mandatory in many countries [55]. Effective interventions should be implemented in the prevention and control of dengue fever. For example, the release of Wolbachia-infected mosquitoes in Indonesia has demonstrated 86% efficacy in community-based interventions, significantly reducing dengue cases [56]. Community mobilization programs in Nicaragua and Mexico have also successfully decreased dengue incidence rates by engaging community participation in education and environmental management measures to reduce mosquito breeding sites [57]. In Guangdong, China, weekly insecticide spraying and patient isolation measures have significantly curtailed dengue transmission, reducing the basic reproduction number from 1.74 to 0.17 [58]. However, ineffective interventions can waste health resources and increase the dengue burden. For instance, the indiscriminate use of CYD-TDV (Dengvaxia) vaccine in individuals who have never been infected with dengue can induce ADE, increasing the risk of severe disease [59]. The long-term use of a single chemical insecticide (e.g., pyrethroids) in *Aedes aegypti* populations has led to increased resistance [60], diminishing the efficacy of insecticides. Additionally, passive interventions that rely solely on government-led insecticide spraying, without community participation or environmental management, have limited effectiveness [61].

This study has several limitations. First, the accuracy and robustness of GBD estimates depend on the quality and quantity of data used in modeling. For countries without systematic dengue surveillance, estimates of dengue – related indicators may be biased. Additionally, differences in diagnostic quality, laboratory standards, and reporting criteria across countries result in significant heterogeneity, potentially affecting the validity of the results. Second, the study lacks data on dengue virus genotypes for countries, preventing an analysis of their relationship with dengue epidemiology. Despite these limitations, the study offers valuable evidence and novel approaches for global dengue prevention and control, enhancing existing prevention and surveillance efforts. Using multiple modeling methods, the study analyzed dengue – related data at global, regional, and national levels, focusing on different genders and age groups. This approach identified key populations for dengue prevention and control, examined health inequalities across regions, and provided targeted suggestions for improving the effectiveness of interventions.

## Conclusion

In conclusion, dengue’s escalating cases and uneven global spread pose significant challenges for disease control. This study’s insights could aid in crafting tailored, adaptable public health policies and resource allocation, crucial for policymakers to enhance personalized healthcare systems. Future research should focus on vaccine development and climate change, with the integration of multiple disciplines, to improve prevention and control strategies and address new global challenges.

## Supporting information

S1 Table The case number and ASR of incidence of Dengue in 1990 and 2021 for both sexes by SDI quintiles and by GBD regions. ASR, age-standardized rate; EAPC, estimated annual percentage change; UIs, uncertainty intervals; CI, confidence interval.
(DOCX)

S2 Table The case number and ASR of prevalence of Dengue in 1990 and 2021 for both sexes by SDI quintiles and by GBD regions. ASR, age-standardized rate; EAPC, estimated annual percentage change; UIs, uncertainty intervals; CI, confidence interval.
(DOCX)

S3 Table The case number and ASR of DALYs of dengue in 1990 and 2021 for both sexes by SDI quintiles and by GBD regions. ASR, age-standardized rate; EAPC, estimated annual percentage change; UIs, uncertainty intervals; CI, confidence interval.
(DOCX)

S4 Table The case number and ASR of deaths of Dengue in 1990 and 2021 for both sexes by SDI quintiles and by GBD regions. ASR, age-standardized rate; EAPC, estimated annual percentage change; UIs, uncertainty intervals; CI, confidence interval.
(DOCX)

S5 Table RRs of Dengue incidence and mortality for both sexes due to age, period, and birth effects (1). RR, relative risks; CI, confidence interval.
(DOCX)

S6 Table RRs of Dengue incidence and mortality for both sexes due to age, period, and birth effects (2). RR, relative risks; CI, confidence interval.
(DOCX)

S7 Table Changes in DALYs of Dengue according to disease categories and population-level determinants from 1990 to 2021. DALYs, disability-adjusted life-years; SDI, socio-demographic index.
(DOCX)

S8 Table The predicted ASR of incidence, prevalence, DALYs and mortality of dengue from 2022 to 2051 globally. ASR, age-standardized rate; DALYs, disability-adjusted life-years; CrI, credible interval.
(DOCX)
