# Supplementary material for: Trends and cross-country inequalities in dengue, 1990–2021
Source: PLoS One. 2025 Jun 20;20(6):e0316694. doi: 10.1371/journal.pone.0316694 (PMC12180626; doi:10.1371/journal.pone.0316694)
Supplement: S1 Table — ASR, age-standardized rate; EAPC, estimated annual percentage change; UIs, uncertainty intervals; CI, confidence interval. (DOCX) [file pone.0316694.s001.docx]

# Table S1. The case number and ASR of incidence of Dengue in 1990 and 2021 for both sexes by SDI quintiles and by GBD regions.

| Location | 1990 | | 2021 | | EAPC_CI/ASIR(%) |
| --- | --- | --- | --- | --- | --- |
|  | Numeber(95%UIs) | ASR(95%UIs) | Numeber(95%UIs) | ASR(95%UIs) |  |
| Global | 26447129 (3933186 to 51891942) | 481.85 (70.76 to 946.29) | 58964185 (15473439 to 106885036) | 752.04 (196.33 to 1363.35) | 1.83 (1.58 to 2.08) |
| High SDI | 324498 (46668 to 790585) | 38.03 (5.47 to 92.83) | 601233 (132601 to 1314069) | 54.64 (12.22 to 119.21) | 1.89 (0.93 to 2.87) |
| High-middle SDI | 1046512 (130035 to 2806272) | 98.43 (12.21 to 263.08) | 2494216 (1061802 to 4307562) | 215.66 (91.61 to 372.3) | 3.38 (2.98 to 3.79) |
| Middle SDI | 13612466 (890230 to 29939998) | 782.97 (51.81 to 1725.4) | 30664157 (10546524 to 54885088) | 1269.27 (437.36 to 2268) | 2.13 (1.83 to 2.43) |
| Low-middle SDI | 9294747  (949482 to  19993512) | 802.75  (79.39 to  1725.04) | 21251661  (2294597 to  45470002) | 1117.7  (123.23 to  2373.55) | 1.37  (1.18 to 1.56) |
| Low-middle SDI | 9294747 (949482 to 19993512) | 802.75 (79.39 to 1725.04) | 21251661 (2294597 to 45470002) | 1117.7 (123.23 to 2373.55) | 1.37 (1.18 to 1.56) |
| Low SDI | 2157287 (1251605 to 3202640) | 431.07 (242.88 to 648.51) | 3932556 (125886 to 9580301) | 368.03 (11.69 to 884.71) | -1.07 (-1.71 to -0.42) |
| Andean Latin America | 117506 (13807 to 275359) | 305.85 (36.08 to 714.51) | 391708 (163083 to 669824) | 593.22 (247.03 to 1014.24) | 2.31 (1.92 to 2.7) |
| Australasia | 5792 (403 to 17086) | 28.69 (2 to 84.63) | 18448 (5642 to 43237) | 58.99 (18.09 to 139.42) | 3.76 (2.93 to 4.59) |
| Caribbean | 145496 (4621 to 426127) | 416.07 (13.34 to 1220.54) | 227073 (29104 to 644226) | 475.93 (60.68 to 1325.33) | 0.65 (-0.02 to 1.33) |
| Central Asia | 0 (0 to 0) | 0 (0 to 0) | 0 (0 to 0) | 0 (0 to 0) | #N/A |
| Central Europe | 0 (0 to 0) | 0 (0 to 0) | 0 (0 to 0) | 0 (0 to 0) | #N/A |
| Central Latin America | 1146583 (190138 to 2317017) | 696.38 (115.55 to 1409.02) | 2886641 (1757444 to 4048875) | 1140.37 (694.53 to 1599.48) | 2.86 (1.74 to 3.98) |
| Central Sub-Saharan Africa | 72873 (888 to 467312) | 133.39 (1.64 to 850.51) | 245097 (11852 to 1403022) | 178.08 (8.62 to 1019.4) | 1.09 (0.99 to 1.18) |
| East Asia | 35782 (3516 to 119645) | 3.03 (0.3 to 10.15) | 61440 (15720 to 155195) | 4.27 (1.11 to 10.66) | 1.15 (0.99 to 1.3) |
| Eastern Europe | 0 (0 to 0) | 0 (0 to 0) | 0 (0 to 0) | 0 (0 to 0) | #N/A |
| Eastern Sub-Saharan Africa | 1586665 (121294 to 4159410) | 829.74 (63.78 to 2162.22) | 387627 (6547 to 1462386) | 94.37 (1.55 to 364.39) | -9.06 (-11.01 to -7.06) |
| High-income Asia Pacific | 219058 (32182 to 572883) | 130.09 (19.12 to 340.2) | 485712 (107597 to 1123586) | 294.01 (65.07 to 679.53) | 3.5 (2.34 to 4.68) |
| High-income North America | 290 (48 to 985) | 0.1 (0.02 to 0.35) | 1376 (40 to 7207) | 0.36 (0.01 to 1.86) | 6.75 (5.3 to 8.22) |
| North Africa and Middle East | 17978 (5946 to 56488) | 5.23 (1.74 to 16.53) | 53391 (17761 to 188663) | 8.5 (2.86 to 30.02) | 1.82 (0.88 to 2.76) |
| Oceania | 24034 (5327 to 63232) | 370.69 (81.9 to 969.58) | 63970 (28314 to 126070) | 486.03 (211.17 to 953.78) | 1.54 (1.19 to 1.88) |
| South Asia | 12693595 (136546 to 27050061) | 1163.51 (12.61 to 2478.46) | 31812189 (1872477 to 67070659) | 1726.94 (102.48 to 3635.94) | 1.48 (1.42 to 1.54) |
| Southeast Asia | 2757882 (539807 to 7562135) | 584.49 (114.66 to 1585.42) | 6728444 (4787955 to 10431378) | 971.89 (691.33 to 1500.41) | 2.17 (1.87 to 2.47) |
| Southern Latin America | 38200 (802 to 132559) | 77.21 (1.62 to 267.93) | 80129 (18027 to 188076) | 118.83 (26.67 to 278.96) | 1.9 (1.43 to 2.37) |
| Southern Sub-Saharan Africa | 689 (33 to 4553) | 1.23 (0.06 to 8.1) | 1087 (57 to 7292) | 1.32 (0.07 to 8.83) | -2.22 (-3.06 to -1.38) |
| Tropical Latin America | 6863968 (405330 to 18146519) | 4460.04 (265.15 to 11852.45) | 13043195 (3996126 to 26305622) | 5774.82 (1774.73 to 11624.76) | 1.59 (1.08 to 2.11) |
| Western Europe | 0 (0 to 0) | 0 (0 to 0) | 0 (0 to 0) | 0 (0 to 0) | #N/A |
| Western Sub-Saharan Africa | 720738 (435 to 2626893) | 378.12 (0.22 to 1357.39) | 2476656 (120934 to 8632875) | 512.53 (25.35 to 1792.58) | 1.08 (0.98 to 1.18) |

**Abbreviations:** ASR, age-standardized rate; EAPC, estimated annual percentage change; UIs, uncertainty intervals; CI, confidence interval.
