# Supplementary material for: Trends and cross-country inequalities in dengue, 1990–2021
Source: PLoS One. 2025 Jun 20;20(6):e0316694. doi: 10.1371/journal.pone.0316694 (PMC12180626; doi:10.1371/journal.pone.0316694)
Supplement: S2 Table — ASR, age-standardized rate; EAPC, estimated annual percentage change; UIs, uncertainty intervals; CI, confidence interval. (DOCX) [file pone.0316694.s002.docx]

# Table S2. The case number and ASR of prevalence of Dengue in 1990 and 2021 for both sexes by SDI quintiles and by GBD regions.

| Location | 1990 | | 2021 | | EAPC_CI/ASPR(%) |
| --- | --- | --- | --- | --- | --- |
|  | Numeber(95%UIs) | ASR(95%UIs) | Numeber(95%UIs) | ASR(95%UIs) |  |
| Global | 1577834 (234723 to 3169007) | 28.75 (4.23 to 57.77) | 3517384 (928244 to 6430039) | 44.86 (11.77 to 82.13) | 1.83 (1.58 to 2.08) |
| High SDI | 19358 (2513 to 49166) | 2.27 (0.29 to 5.75) | 35973 (7568 to 83314) | 3.27 (0.69 to 7.59) | 1.9 (0.94 to 2.88) |
| High-middle SDI | 62126 (7361 to 172340) | 5.84 (0.69 to 16.18) | 147982 (58601 to 266672) | 12.79 (5.04 to 23.03) | 3.4 (2.98 to 3.81) |
| Middle SDI | 812124 (52359 to 1773434) | 46.71 (3.05 to 102.3) | 1828908 (616235 to 3309026) | 75.7 (25.59 to 136.59) | 2.13 (1.83 to 2.43) |
| Low-middle SDI | 555210 (56563 to 1176069) | 47.95 (4.73 to 101.66) | 1269643 (137243 to 2706196) | 66.77 (7.37 to 141.34) | 1.37 (1.18 to 1.56) |
| Low SDI | 128325 (71919 to 193957) | 25.64 (14.03 to 39.36) | 233663 (7366 to 592775) | 21.87 (0.68 to 54.56) | -1.08 (-1.73 to -0.43) |
| Andean Latin America | 7040 (802 to 16841) | 18.32 (2.1 to 43.68) | 23429 (9709 to 41698) | 35.48 (14.7 to 63.09) | 2.3 (1.92 to 2.69) |
| Australasia | 346 (24 to 1108) | 1.71 (0.12 to 5.49) | 1103 (304 to 2901) | 3.53 (0.97 to 9.25) | 3.77 (2.94 to 4.61) |
| Caribbean | 8670 (268 to 26126) | 24.79 (0.77 to 74.65) | 13594 (1664 to 38337) | 28.49 (3.49 to 80.01) | 0.66 (-0.01 to 1.34) |
| Central Asia | 0 (0 to 0) | 0 (0 to 0) | 0 (0 to 0) | 0 (0 to 0) | #N/A |
| Central Europe | 0 (0 to 0) | 0 (0 to 0) | 0 (0 to 0) | 0 (0 to 0) | #N/A |
| Central Latin America | 68341 (11324 to 139651) | 41.51 (6.89 to 85.01) | 172010 (102576 to 247213) | 67.95 (40.54 to 97.63) | 2.86 (1.74 to 3.99) |
| Central Sub-Saharan Africa | 4379 (53 to 24980) | 8.02 (0.1 to 45.52) | 14947 (677 to 81024) | 10.86 (0.5 to 59.03) | 1.07 (0.99 to 1.14) |
| East Asia | 2124 (211 to 6597) | 0.18 (0.02 to 0.56) | 3637 (918 to 9167) | 0.25 (0.06 to 0.62) | 1.13 (0.98 to 1.28) |
| Eastern Europe | 0 (0 to 0) | 0 (0 to 0) | 0 (0 to 0) | 0 (0 to 0) | #N/A |
| Eastern Sub-Saharan Africa | 94477 (7197 to 255561) | 49.41 (3.77 to 133.14) | 22953 (372 to 88355) | 5.59 (0.09 to 21.71) | -9.08 (-11.03 to -7.08) |
| High-income Asia Pacific | 13085 (1800 to 35761) | 7.77 (1.07 to 21.24) | 29078 (5850 to 71642) | 17.6 (3.54 to 43.36) | 3.51 (2.35 to 4.69) |
| High-income North America | 17 (3 to 58) | 0.01 (0 to 0.02) | 80 (2 to 402) | 0.02 (0 to 0.1) | 6.79 (5.33 to 8.27) |
| North Africa and Middle East | 1071 (358 to 3424) | 0.31 (0.1 to 1) | 3193 (1042 to 11473) | 0.51 (0.17 to 1.83) | 1.81 (0.86 to 2.76) |
| Oceania | 1425 (314 to 3750) | 21.98 (4.85 to 56.47) | 3811 (1670 to 7579) | 28.95 (12.35 to 58.06) | 1.54 (1.19 to 1.88) |
| South Asia | 759103 (7932 to 1648564) | 69.58 (0.73 to 150.95) | 1901418 (107574 to 4067253) | 103.22 (5.9 to 220.59) | 1.48 (1.41 to 1.54) |
| Southeast Asia | 163793 (31414 to 458791) | 34.71 (6.68 to 96.33) | 400005 (281601 to 633455) | 57.78 (40.63 to 91.25) | 2.18 (1.88 to 2.48) |
| Southern Latin America | 2269 (45 to 8031) | 4.59 (0.09 to 16.23) | 4765 (1007 to 11401) | 7.07 (1.49 to 16.92) | 1.91 (1.44 to 2.39) |
| Southern Sub-Saharan Africa | 42 (2 to 283) | 0.07 (0 to 0.5) | 63 (3 to 428) | 0.08 (0 to 0.52) | -2.3 (-3.14 to -1.46) |
| Tropical Latin America | 408763 (24011 to 1056404) | 265.61 (15.64 to 691.39) | 776465 (243162 to 1587515) | 343.77 (107.96 to 700.31) | 1.59 (1.08 to 2.1) |
| Western Europe | 0 (0 to 0) | 0 (0 to 0) | 0 (0 to 0) | 0 (0 to 0) | #N/A |
| Western Sub-Saharan Africa | 42888 (26 to 148118) | 22.5 (0.01 to 78.76) | 146832 (7218 to 530938) | 30.38 (1.51 to 111.89) | 1.07 (0.98 to 1.16) |

**Abbreviations:** ASR, age-standardized rate; EAPC, estimated annual percentage change; UIs, uncertainty intervals; CI, confidence interval.
