# Supplementary material for: Trends and cross-country inequalities in dengue, 1990–2021
Source: PLoS One. 2025 Jun 20;20(6):e0316694. doi: 10.1371/journal.pone.0316694 (PMC12180626; doi:10.1371/journal.pone.0316694)
Supplement: S3 Table — ASR, age-standardized rate; EAPC, estimated annual percentage change; UIs, uncertainty intervals; CI, confidence interval. (DOCX) [file pone.0316694.s003.docx]

# Table S3. The case number and ASR of DALYs of Dengue in 1990 and 2021 for both sexes by SDI quintiles and by GBD regions.

| Location | 1990 | | 2021 | | EAPC_CI/ASDR(%) |
| --- | --- | --- | --- | --- | --- |
|  | Numeber(95%UIs) | ASR(95%UIs) | Numeber(95%UIs) | ASR(95%UIs) |  |
| Global | 1248669 (876050 to 1552996) | 21.63 (15.09 to 26.92) | 2076525 (1056228 to 3130718) | 27.76 (14.21 to 41.65) | 1.33 (1.1 to 1.57) |
| High SDI | 3540 (698 to 9047) | 0.42 (0.09 to 1.07) | 6495 (1607 to 15294) | 0.58 (0.14 to 1.39) | 1.96 (1.04 to 2.9) |
| High-middle SDI | 87624 (61327 to 121293) | 8.92 (6.2 to 12.61) | 110148 (64063 to 156180) | 11.39 (6.67 to 16.03) | 1 (0.67 to 1.33) |
| Middle SDI | 630453 (403745 to 805798) | 33.64 (21.46 to 42.85) | 1045828 (575252 to 1544204) | 48.78 (27.32 to 71.02) | 1.87 (1.58 to 2.16) |
| Low-middle SDI | 463610 (326499 to 593003) | 35.92 (24.95 to 47.94) | 793856 (351112 to 1290420) | 43.35 (19.69 to 69.74) | 1.06 (0.86 to 1.25) |
| Low SDI | 62236 (43902 to 85212) | 11.84 (8.11 to 16.08) | 118795 (38239 to 222378) | 12.34 (4.44 to 22.08) | 0.34 (0.05 to 0.64) |
| Andean Latin America | 1423 (304 to 3501) | 3.58 (0.74 to 8.91) | 4327 (1845 to 8129) | 6.56 (2.8 to 12.31) | 2.58 (1.69 to 3.47) |
| Australasia | 60 (4 to 200) | 0.3 (0.02 to 0.99) | 192 (50 to 498) | 0.61 (0.16 to 1.6) | 3.81 (2.97 to 4.65) |
| Caribbean | 1785 (304 to 5236) | 5.02 (0.84 to 14.8) | 2751 (546 to 7217) | 5.83 (1.17 to 15.48) | 1.12 (-0.04 to 2.29) |
| Central Asia | 0 (0 to 0) | 0 (0 to 0) | 0 (0 to 0) | 0 (0 to 0) | #N/A |
| Central Europe | 0 (0 to 0) | 0 (0 to 0) | 0 (0 to 0) | 0 (0 to 0) | #N/A |
| Central Latin America | 14066 (4221 to 29842) | 8.26 (2.37 to 17.68) | 43074 (24433 to 67687) | 17.53 (9.92 to 27.6) | 3.63 (2.67 to 4.59) |
| Central Sub-Saharan Africa | 747 (12 to 4637) | 1.36 (0.02 to 8.41) | 2646 (126 to 14646) | 1.91 (0.09 to 10.31) | 1.14 (1.06 to 1.22) |
| East Asia | 3890 (2247 to 5662) | 0.34 (0.2 to 0.49) | 1360 (568 to 2540) | 0.09 (0.04 to 0.18) | -3.38 (-3.94 to -2.8) |
| Eastern Europe | 0 (0 to 0) | 0 (0 to 0) | 0 (0 to 0) | 0 (0 to 0) | #N/A |
| Eastern Sub-Saharan Africa | 16106 (1433 to 42354) | 8.17 (0.69 to 21.51) | 5941 (1375 to 17523) | 1.28 (0.23 to 4.12) | -7.81 (-9.69 to -5.89) |
| High-income Asia Pacific | 2175 (268 to 5922) | 1.3 (0.17 to 3.53) | 4713 (876 to 12045) | 2.9 (0.52 to 7.39) | 3.5 (2.33 to 4.68) |
| High-income North America | 7 (4 to 14) | 0 (0 to 0.01) | 42 (16 to 121) | 0.02 (0.01 to 0.04) | 8 (7.16 to 8.85) |
| North Africa and Middle East | 895 (498 to 1516) | 0.25 (0.15 to 0.41) | 1035 (445 to 2669) | 0.18 (0.08 to 0.45) | 0.82 (-1.03 to 2.71) |
| Oceania | 946 (645 to 1424) | 13.43 (8.6 to 20.83) | 767 (347 to 1540) | 6.04 (2.73 to 11.88) | -0.02 (-0.74 to 0.71) |
| South Asia | 388994 (197761 to 625033) | 35.79 (17.97 to 57.41) | 931668 (324113 to 1630477) | 53.46 (19.54 to 91.91) | 1.88 (1.64 to 2.12) |
| Southeast Asia | 742194 (469882 to 1158430) | 134.99 (88.76 to 204.74) | 909114 (589035 to 1241631) | 147.04 (95.32 to 200.97) | 0.74 (0.54 to 0.94) |
| Southern Latin America | 400 (15 to 1509) | 0.81 (0.03 to 3.05) | 820 (150 to 2211) | 1.22 (0.22 to 3.28) | 1.8 (1.33 to 2.28) |
| Southern Sub-Saharan Africa | 9 (1 to 50) | 0.02 (0 to 0.09) | 12 (1 to 79) | 0.01 (0 to 0.1) | -2.21 (-3.01 to -1.42) |
| Tropical Latin America | 67757 (5126 to 176965) | 43.56 (3.32 to 113.62) | 143870 (50995 to 306624) | 63.76 (22.43 to 136.04) | 2.23 (1.69 to 2.76) |
| Western Europe | 91 (43 to 158) | 0.02 (0.01 to 0.03) | 1 (1 to 3) | 0 (0 to 0) | -7.95 (-12.28 to -3.41) |
| Western Sub-Saharan Africa | 7124 (60 to 27505) | 3.68 (0.02 to 14.27) | 24191 (1157 to 83576) | 4.93 (0.24 to 17.05) | 1.08 (0.99 to 1.17) |

**Abbreviations:** ASR, age-standardized rate; EAPC, estimated annual percentage change; UIs, uncertainty intervals; CI, confidence interval.
