# Supplementary material for: Trends and cross-country inequalities in dengue, 1990–2021
Source: PLoS One. 2025 Jun 20;20(6):e0316694. doi: 10.1371/journal.pone.0316694 (PMC12180626; doi:10.1371/journal.pone.0316694)
Supplement: S4 Table — ASR, age-standardized rate; EAPC, estimated annual percentage change; UIs, uncertainty intervals; CI, confidence interval. (DOCX) [file pone.0316694.s004.docx]

# Table S4. The case number and ASR of deaths of Dengue in 1990 and 2021 for both sexes by SDI quintiles and by GBD regions.

| Location | 1990 | | 2021 | | EAPC_CI/ASMR(%) |
| --- | --- | --- | --- | --- | --- |
|  | Numeber(95%UIs) | ASR(95%UIs) | Numeber(95%UIs) | ASR(95%UIs) |  |
| Global | 14315 (11103 to 18652) | 0.27 (0.21 to 0.33) | 29076 (17628 to 38980) | 0.38 (0.23 to 0.51) | 1.7 (1.45 to 1.94) |
| High SDI | 15 (9 to 22) | 0 (0 to 0) | 31 (14 to 50) | 0 (0 to 0) | 4.37 (2.66 to 6.1) |
| High-middle SDI | 1058 (773 to 1505) | 0.11 (0.08 to 0.16) | 1549 (930 to 2111) | 0.14 (0.08 to 0.19) | 0.94 (0.62 to 1.26) |
| Middle SDI | 6766 (4827 to 9848) | 0.4 (0.31 to 0.55) | 13065 (8154 to 17810) | 0.6 (0.37 to 0.81) | 1.98 (1.69 to 2.27) |
| Low-middle SDI | 5782 (4471 to 7165) | 0.59 (0.47 to 0.72) | 12681 (7342 to 17711) | 0.84 (0.5 to 1.18) | 1.75 (1.51 to 1.99) |
| Low SDI | 681 (484 to 948) | 0.2 (0.14 to 0.28) | 1733 (848 to 2766) | 0.3 (0.17 to 0.46) | 2.04 (1.7 to 2.38) |
| Andean Latin America | 4 (2 to 6) | 0.01 (0.01 to 0.01) | 11 (5 to 17) | 0.02 (0.01 to 0.03) | 3.44 (1.08 to 5.85) |
| Australasia | 0 (0 to 0) | 0 (0 to 0) | 0 (0 to 0) | 0 (0 to 0) | 7.76 (6.3 to 9.24) |
| Caribbean | 6 (4 to 8) | 0.02 (0.01 to 0.02) | 12 (6 to 20) | 0.02 (0.01 to 0.04) | 2.38 (0.3 to 4.52) |
| Central Asia | 0 (0 to 0) | 0 (0 to 0) | 0 (0 to 0) | 0 (0 to 0) | #N/A |
| Central Europe | 0 (0 to 0) | 0 (0 to 0) | 0 (0 to 0) | 0 (0 to 0) | #N/A |
| Central Latin America | 44 (34 to 52) | 0.03 (0.02 to 0.04) | 292 (161 to 457) | 0.12 (0.07 to 0.2) | 5.51 (4.07 to 6.96) |
| Central Sub-Saharan Africa | 0 (0 to 0) | 0 (0 to 0) | 1 (0 to 1) | 0 (0 to 0) | 1.71 (1.49 to 1.93) |
| East Asia | 46 (28 to 65) | 0 (0 to 0.01) | 30 (14 to 49) | 0 (0 to 0) | -0.66 (-1.87 to 0.57) |
| Eastern Europe | 0 (0 to 0) | 0 (0 to 0) | 0 (0 to 0) | 0 (0 to 0) | #N/A |
| Eastern Sub-Saharan Africa | 9 (3 to 19) | 0 (0 to 0.01) | 27 (15 to 49) | 0.01 (0 to 0.01) | 1.4 (1.06 to 1.74) |
| High-income Asia Pacific | 1 (0 to 1) | 0 (0 to 0) | 1 (1 to 2) | 0 (0 to 0) | 1.11 (0.09 to 2.15) |
| High-income North America | 0 (0 to 0) | 0 (0 to 0) | 1 (0 to 1) | 0 (0 to 0) | 8.61 (7.59 to 9.63) |
| North Africa and Middle East | 13 (7 to 20) | 0.01 (0 to 0.01) | 16 (8 to 27) | 0 (0 to 0.01) | 0.12 (-1.39 to 1.66) |
| Oceania | 11 (8 to 15) | 0.23 (0.16 to 0.34) | 4 (2 to 6) | 0.05 (0.02 to 0.1) | -1.96 (-2.82 to -1.09) |
| South Asia | 4802 (3450 to 6453) | 0.64 (0.46 to 0.86) | 15447 (8464 to 22362) | 1.08 (0.62 to 1.54) | 2.45 (2.12 to 2.78) |
| Southeast Asia | 9340 (6061 to 14351) | 1.87 (1.3 to 2.7) | 12675 (7893 to 17449) | 2.04 (1.28 to 2.82) | 0.69 (0.49 to 0.89) |
| Southern Latin America | 0 (0 to 0) | 0 (0 to 0) | 0 (0 to 0) | 0 (0 to 0) | -9.14 (-10.18 to -8.09) |
| Southern Sub-Saharan Africa | 0 (0 to 0) | 0 (0 to 0) | 0 (0 to 0) | 0 (0 to 0) | -2.08 (-2.59 to -1.56) |
| Tropical Latin America | 28 (20 to 39) | 0.02 (0.02 to 0.03) | 558 (353 to 746) | 0.23 (0.15 to 0.31) | 10.79 (9.23 to 12.37) |
| Western Europe | 10 (5 to 17) | 0 (0 to 0) | 0 (0 to 0) | 0 (0 to 0) | -8.71 (-14.16 to -2.91) |
| Western Sub-Saharan Africa | 1 (1 to 2) | 0 (0 to 0) | 2 (1 to 4) | 0 (0 to 0) | 0.69 (-0.76 to 2.16) |

**Abbreviations:** ASR, age-standardized rate; EAPC, estimated annual percentage change; UIs, uncertainty intervals; CI, confidence interval.
