# Supplementary material for: Trends and cross-country inequalities in dengue, 1990–2021
Source: PLoS One. 2025 Jun 20;20(6):e0316694. doi: 10.1371/journal.pone.0316694 (PMC12180626; doi:10.1371/journal.pone.0316694)
Supplement: S5 Table — RR, relative risks; CI, confidence interval. (DOCX) [file pone.0316694.s005.docx]

# Table S5. RRs of Dengue incidence and mortality for both sexes due to age,period,and birth effects (1).

| Factor | Incidence | | Mortality | | Factor | Incidence | | Mortality | | |
| --- | --- | --- | --- | --- | --- | --- | --- | --- | --- | --- |
|  | RR(95%CI) | *P* | RR(95%CI) | *P* |  | RR(95%CI) | *P* | RR(95%CI) | *P* | |
| Age(years) |  |  |  |  | Local drifts |  |  |  |  | |
| 2.5 | 44567.276 (31497.653 to 63060.003) | <0.001 | 44567.276 (31497.653 to 63060.003) | <0.001 | 2.5 | 0.729 (-1.226 to 2.722) | <0.001 | 0.729 (-1.226 to 2.722) | <0.001 | |
| 7.5 | 225.486 (221.541 to 229.501) | <0.001 | 225.486 (221.541 to 229.501) | <0.001 | 7.5 | 1.129 (1.068 to 1.19) | <0.001 | 1.129 (1.068 to 1.19) | <0.001 | |
| 12.5 | 408.658 (402.12 to 415.301) | <0.001 | 408.658 (402.12 to 415.301) | <0.001 | 12.5 | 1.238 (1.196 to 1.281) | <0.001 | 1.238 (1.196 to 1.281) | <0.001 | |
| 17.5 | 450.649 (443.645 to 457.762) | <0.001 | 450.649 (443.645 to 457.762) | <0.001 | 17.5 | 1.421 (1.383 to 1.459) | <0.001 | 1.421 (1.383 to 1.459) | <0.001 | |
| 22.5 | 444.228 (437.421 to 451.142) | <0.001 | 444.228 (437.421 to 451.142) | <0.001 | 22.5 | 1.722 (1.685 to 1.76) | <0.001 | 1.722 (1.685 to 1.76) | <0.001 | |
| 27.5 | 446.588 (439.834 to 453.447) | <0.001 | 446.588 (439.834 to 453.447) | <0.001 | 27.5 | 1.915 (1.876 to 1.955) | <0.001 | 1.915 (1.876 to 1.955) | <0.001 | |
| 32.5 | 453.775 (447.001 to 460.651) | <0.001 | 453.775 (447.001 to 460.651) | <0.001 | 32.5 | 1.92 (1.878 to 1.962) | <0.001 | 1.92 (1.878 to 1.962) | <0.001 | |
| 37.5 | 449.903 (443.514 to 456.384) | <0.001 | 449.903 (443.514 to 456.384) | <0.001 | 37.5 | 1.973 (1.928 to 2.018) | <0.001 | 1.973 (1.928 to 2.018) | <0.001 | |
| 42.5 | 475.769 (469.09 to 482.543) | <0.001 | 475.769 (469.09 to 482.543) | <0.001 | 42.5 | 2.14 (2.09 to 2.189) | <0.001 | 2.14 (2.09 to 2.189) | <0.001 | |
| 47.5 | 478.94 (472.12 to 485.858) | <0.001 | 478.94 (472.12 to 485.858) | <0.001 | 47.5 | 2.068 (2.014 to 2.123) | <0.001 | 2.068 (2.014 to 2.123) | <0.001 | |
| 52.5 | 491.507 (484.307 to 498.814) | <0.001 | 491.507 (484.307 to 498.814) | <0.001 | 52.5 | 1.91 (1.85 to 1.97) | <0.001 | 1.91 (1.85 to 1.97) | <0.001 | |
| 57.5 | 543.253 (535.13 to 551.499) | <0.001 | 543.253 (535.13 to 551.499) | <0.001 | 57.5 | 1.969 (1.903 to 2.035) | <0.001 | 1.969 (1.903 to 2.035) | <0.001 | |
| 62.5 | 592.748 (583.525 to 602.116) | <0.001 | 592.748 (583.525 to 602.116) | <0.001 | 62.5 | 2.111 (2.038 to 2.184) | <0.001 | 2.111 (2.038 to 2.184) | <0.001 | |
| 67.5 | 684.328 (672.815 to 696.038) | <0.001 | 684.328 (672.815 to 696.038) | <0.001 | 67.5 | 2.248 (2.166 to 2.33) | <0.001 | 2.248 (2.166 to 2.33) | <0.001 | |
| 72.5 | 753.884 (738.482 to 769.607) | <0.001 | 753.884 (738.482 to 769.607) | <0.001 | 72.5 | 2.378 (2.283 to 2.474) | <0.001 | 2.378 (2.283 to 2.474) | <0.001 | |
| 77.5 | 821.099 (801.946 to 840.71) | <0.001 | 821.099 (801.946 to 840.71) | <0.001 | 77.5 | 2.411 (2.294 to 2.527) | <0.001 | 2.411 (2.294 to 2.527) | <0.001 |  |
| 82.5 | 957.644 (931.99 to 984.004) | <0.001 | 957.644 (931.99 to 984.004) | <0.001 | 82.5 | 2.482 (2.336 to 2.629) | <0.001 | 2.482 (2.336 to 2.629) | <0.001 | |
| 87.5 | 1022.566 (988.657 to 1057.637) | <0.001 | 1022.566 (988.657 to 1057.637) | <0.001 | 87.5 | 2.471 (2.277 to 2.665) | <0.001 | 2.471 (2.277 to 2.665) | <0.001 | |
| 92.5 | 1350.948 (1294.201 to 1410.183) | <0.001 | 1350.948 (1294.201 to 1410.183) | <0.001 | 92.5 | 2.607 (2.328 to 2.887) | <0.001 | 2.607 (2.328 to 2.887) | <0.001 | |
| 97.5 | 2715.626 (2574.64 to 2864.333) | <0.001 | 2715.626 (2574.64 to 2864.333) | <0.001 | 97.5 | 2.87 (2.433 to 3.308) | <0.001 | 2.87 (2.433 to 3.308) | <0.001 | |
| 102.5 | 8367.372 (7864.782 to 8902.079) | <0.001 | 8367.372 (7864.782 to 8902.079) | <0.001 | 102.5 | 3.367 (2.604 to 4.135) | <0.001 | 3.367 (2.604 to 4.135) | <0.001 | |

Abbreviations: RR, relative risks; CI, confidence interval.
