# Supplementary material for: Trends and cross-country inequalities in dengue, 1990–2021
Source: PLoS One. 2025 Jun 20;20(6):e0316694. doi: 10.1371/journal.pone.0316694 (PMC12180626; doi:10.1371/journal.pone.0316694)
Supplement: S6 Table — RR, relative risks; CI, confidence interval. (DOCX) [file pone.0316694.s006.docx]

# Table S6. RRs of Dengue incidence and mortality for both sexes due to age,period,and birth effects (2).

| Factor | Incidence | | Mortality | |
| --- | --- | --- | --- | --- |
|  | RR(95%CI) | *P* | RR(95%CI) | *P* |
| Period |  |  |  |  |
| 1994.5 | 0.806 (0.796 to 0.816) | <0.001 | 0.806 (0.796 to 0.816) | <0.001 |
| 1999.5 | 0.88 (0.871 to 0.889) | <0.001 | 0.88 (0.871 to 0.889) | <0.001 |
| 2004.5 | 0.918 (0.91 to 0.927) | <0.001 | 0.918 (0.91 to 0.927) | <0.001 |
| 2009.5 | 1 (1 to 1) | <0.001 | 1 (1 to 1) | <0.001 |
| 2014.5 | 1.192 (1.182 to 1.202) | <0.001 | 1.192 (1.182 to 1.202) | <0.001 |
| 2019.5 | 1.459 (1.445 to 1.473) | <0.001 | 1.459 (1.445 to 1.473) | <0.001 |
| 2024.5 | 1.369 (1.353 to 1.384) | <0.001 | 1.369 (1.353 to 1.384) | <0.001 |
| Birth cohort |  | <0.001 |  | <0.001 |
| 1892 | 0.164 (0.119 to 0.226) | <0.001 | 0.164 (0.119 to 0.226) | <0.001 |
| 1897 | 0.211 (0.176 to 0.252) | <0.001 | 0.211 (0.176 to 0.252) | <0.001 |
| 1902 | 0.258 (0.231 to 0.289) | <0.001 | 0.258 (0.231 to 0.289) | <0.001 |
| 1907 | 0.309 (0.286 to 0.333) | <0.001 | 0.309 (0.286 to 0.333) | <0.001 |
| 1912 | 0.354 (0.335 to 0.374) | <0.001 | 0.354 (0.335 to 0.374) | <0.001 |
| 1917 | 0.418 (0.401 to 0.437) | <0.001 | 0.418 (0.401 to 0.437) | <0.001 |
| 1922 | 0.439 (0.424 to 0.455) | <0.001 | 0.439 (0.424 to 0.455) | <0.001 |
| 1927 | 0.5 (0.486 to 0.515) | <0.001 | 0.5 (0.486 to 0.515) | <0.001 |
| 1932 | 0.579 (0.565 to 0.594) | <0.001 | 0.579 (0.565 to 0.594) | <0.001 |
| 1937 | 0.655 (0.641 to 0.669) | <0.001 | 0.655 (0.641 to 0.669) | <0.001 |
| 1942 | 0.752 (0.738 to 0.767) | <0.001 | 0.752 (0.738 to 0.767) | <0.001 |
| 1947 | 0.812 (0.798 to 0.827) | <0.001 | 0.812 (0.798 to 0.827) | <0.001 |
| 1952 | 0.873 (0.859 to 0.887) | <0.001 | 0.873 (0.859 to 0.887) | <0.001 |
| 1957 | 1 (1 to 1) | <0.001 | 1 (1 to 1) | <0.001 |
| 1962 | 1.102 (1.086 to 1.118) | <0.001 | 1.102 (1.086 to 1.118) | <0.001 |
| 1967 | 1.177 (1.16 to 1.194) | <0.001 | 1.177 (1.16 to 1.194) | <0.001 |
| 1972 | 1.314 (1.295 to 1.334) | <0.001 | 1.314 (1.295 to 1.334) | <0.001 |
| 1977 | 1.522 (1.5 to 1.545) | <0.001 | 1.522 (1.5 to 1.545) | <0.001 |
| 1982 | 1.671 (1.646 to 1.696) | <0.001 | 1.671 (1.646 to 1.696) | <0.001 |
| 1987 | 1.731 (1.704 to 1.758) | <0.001 | 1.731 (1.704 to 1.758) | <0.001 |
| 1992 | 1.91 (1.88 to 1.941) | <0.001 | 1.91 (1.88 to 1.941) | <0.001 |
| 1997 | 2.131 (2.096 to 2.166) | <0.001 | 2.131 (2.096 to 2.166) | <0.001 |
| 2002 | 2.229 (2.191 to 2.267) | <0.001 | 2.229 (2.191 to 2.267) | <0.001 |
| 2007 | 2.264 (2.224 to 2.305) | <0.001 | 2.264 (2.224 to 2.305) | <0.001 |
| 2012 | 2.356 (2.309 to 2.404) | <0.001 | 2.356 (2.309 to 2.404) | <0.001 |
| 2017 | 2.49 (2.423 to 2.559) | <0.001 | 2.49 (2.423 to 2.559) | <0.001 |
| 2022 | 2.372 (0.951 to 5.917) | <0.001 | 2.372 (0.951 to 5.917) | <0.001 |

Abbreviations: RR, relative risks; CI, confidence interval.
