# Supplementary material for: Trends and cross-country inequalities in dengue, 1990–2021
Source: PLoS One. 2025 Jun 20;20(6):e0316694. doi: 10.1371/journal.pone.0316694 (PMC12180626; doi:10.1371/journal.pone.0316694)
Supplement: S7 Table — DALYs, disability-adjusted life-years; SDI, socio-demographic index. (DOCX) [file pone.0316694.s007.docx]

# Table S7. Changes in DALYs of Dengue according to disease categories and population-level determinants from 1990 to 2021.

| Disease | Location | Overall difference | Aging | Population | Epidemiological change | Aging Percent | Population Percent | Epidemiological change percent |
| --- | --- | --- | --- | --- | --- | --- | --- | --- |
| Dengue-both | Global | 996712.02 | -294508.163 | 745014.688 | 546205.5 | -29.55 | 74.75 | 54.80 |
| Dengue-both | High SDI | 2733.78 | -527.54 | 1351.859 | 1909.463 | -19.3 | 49.45 | 69.85 |
| Dengue-both | High-Midlle SDI | 42486.32 | -1585.279 | 12091.692 | 31979.905 | -3.73 | 28.46 | 75.27 |
| Dengue-both | Middle SDI | 484767.31 | -268721.314 | 357563.782 | 395924.845 | -55.43 | 73.76 | 81.67 |
| Dengue-both | Low-Middle SDI | 391216.38 | 6133.287 | 244581.493 | 140501.605 | 1.57 | 62.52 | 35.91 |
| Dengue-both | Low SDI | 213559.92 | -32781.591 | 181789.861 | 64551.651 | -15.35 | 85.12 | 30.23 |
| Dengue-male | Global | 537116.6 | -180851.27 | 420315.641 | 297652.232 | -33.67 | 78.25 | 55.42 |
| Dengue-male | High SDI | 2050.81 | -219.403 | 649.127 | 1621.087 | -10.7 | 31.65 | 79.05 |
| Dengue-male | High-Midlle SDI | 23958.29 | -516.195 | 6345.24 | 18129.244 | -2.15 | 26.48 | 75.67 |
| Dengue-male | Middle SDI | 263997.71 | -153981.735 | 193363.665 | 224615.781 | -58.33 | 73.24 | 85.08 |
| Dengue-male | Low-Middle SDI | 204374.42 | 2379.649 | 125603.544 | 76391.223 | 1.16 | 61.46 | 37.38 |
| Dengue-male | Low SDI | 122499.47 | -16675.878 | 95469.654 | 43705.693 | -13.61 | 77.93 | 35.68 |
| Dengue-female | Global | 449810.23 | -127205.967 | 341402.374 | 235613.826 | -28.28 | 75.9 | 52.38 |
| Dengue-female | High SDI | 1169.64 | -275.356 | 672.435 | 772.559 | -23.54 | 57.49 | 66.05 |
| Dengue-female | High-Midlle SDI | 18787.13 | -576.335 | 5680.423 | 13683.038 | -3.07 | 30.24 | 72.83 |
| Dengue-female | Middle SDI | 214963.49 | -120780.472 | 168617.48 | 167126.479 | -56.19 | 78.44 | 77.75 |
| Dengue-female | Low-Middle SDI | 187286.34 | 4077.849 | 118805.653 | 64402.841 | 2.18 | 63.44 | 34.39 |
| Dengue-female | Low SDI | 94738.63 | -16404.389 | 87543.397 | 23599.626 | -17.32 | 92.41 | 24.91 |

Abbreviations: DALYs, disability-adjusted life-years; SDI, sociodemographic index.
