# Supplementary material for: Trends and cross-country inequalities in dengue, 1990–2021
Source: PLoS One. 2025 Jun 20;20(6):e0316694. doi: 10.1371/journal.pone.0316694 (PMC12180626; doi:10.1371/journal.pone.0316694)
Supplement: S8 Table — ASR, age-standardized rate; DALYs, disability-adjusted life-years; CrI, credible interval. (DOCX) [file pone.0316694.s008.docx]

# Table S8. The predicted ASR of incidence,prevalence,DALYs and mortality of Dengue from 2022 to 2051 globally.

|  | Incidence | | | Prevalence | | | DALYs | | | Mortality | | |
| --- | --- | --- | --- | --- | --- | --- | --- | --- | --- | --- | --- | --- |
| Year | Sex name | ASR(95% Crl) | Sex name | ASR(95% Crl) | Sex name | ASR(95% Crl) | Sex name | ASR(95% Crl) | Sex name | ASR(95% Crl) | Sex name | ASR(95% Crl) |
| 2022 | Male | 675.01 (642.16 to 707.86) | Female | 788.06 (0.01 to 825.69) | Male | 40.45 (38.64 to 42.25) | Female | 47.1 (44.95 to 49.25) | Male | 28.61 (26.86 to 30.36) | Female | 24.39 (23.01 to 25.77) |
| 2023 | Male | 674.78 (612.33 to 737.23) | Female | 787.95 (0.01 to 862.41) | Male | 40.38 (36.83 to 43.94) | Female | 47.03 (42.69 to 51.37) | Male | 27.86 (25.32 to 30.41) | Female | 23.58 (21.59 to 25.58) |
| 2024 | Male | 674.83 (575.52 to 774.14) | Female | 788.2 (0.01 to 908.26) | Male | 40.34 (34.62 to 46.06) | Female | 46.99 (39.95 to 54.03) | Male | 27.13 (23.57 to 30.69) | Female | 22.8 (20.02 to 25.57) |
| 2025 | Male | 675.02 (533.14 to 816.91) | Female | 788.64 (0.01 to 961.35) | Male | 40.31 (32.1 to 48.52) | Female | 46.96 (36.81 to 57.11) | Male | 26.41 (21.71 to 31.12) | Female | 22.03 (18.39 to 25.67) |
| 2026 | Male | 675.09 (485.72 to 864.46) | Female | 788.88 (0.01 to 1020.3) | Male | 40.27 (29.28 to 51.25) | Female | 46.92 (33.31 to 60.52) | Male | 25.71 (19.77 to 31.66) | Female | 21.29 (16.72 to 25.85) |
| 2027 | Male | 675.05 (433.75 to 916.35) | Female | 788.93 (0 to 1084.58) | Male | 40.21 (26.21 to 54.22) | Female | 46.86 (29.48 to 64.24) | Male | 25.03 (17.79 to 32.27) | Female | 20.57 (15.05 to 26.09) |
| 2028 | Male | 675.2 (377.82 to 972.57) | Female | 789.23 (0 to 1154.31) | Male | 40.17 (22.91 to 57.44) | Female | 46.82 (25.37 to 68.27) | Male | 24.36 (15.79 to 32.93) | Female | 19.87 (13.39 to 26.36) |
| 2029 | Male | 675.59 (318.19 to 1032.99) | Female | 789.86 (0 to 1229.31) | Male | 40.15 (19.4 to 60.91) | Female | 46.8 (20.99 to 72.61) | Male | 23.71 (13.78 to 33.63) | Female | 19.19 (11.74 to 26.65) |
| 2030 | Male | 676.12 (254.98 to 1097.25) | Female | 790.65 (0 to 1309.11) | Male | 40.14 (15.69 to 64.59) | Female | 46.79 (16.36 to 77.22) | Male | 23.07 (11.77 to 34.36) | Female | 18.53 (10.12 to 26.95) |
| 2031 | Male | 676.52 (188.27 to 1164.78) | Female | 791.25 (0 to 1392.93) | Male | 40.12 (11.78 to 68.46) | Female | 46.77 (11.49 to 82.05) | Male | 22.44 (9.78 to 35.11) | Female | 17.9 (8.54 to 27.26) |
| 2032 | Male | 676.83 (118.2 to 1235.46) | Female | 791.67 (0 to 1480.57) | Male | 40.09 (7.69 to 72.49) | Female | 46.73 (6.38 to 87.09) | Male | 21.84 (7.8 to 35.87) | Female | 17.28 (6.99 to 27.56) |
| 2033 | Male | 677.29 (44.98 to 1309.61) | Female | 792.3 (0 to 1572.59) | Male | 40.07 (3.42 to 76.72) | Female | 46.71 (1.05 to 92.38) | Male | 21.25 (5.85 to 36.64) | Female | 16.68 (5.49 to 27.86) |
| 2034 | Male | 677.97 (-31.32 to 1387.25) | Female | 793.21 (0 to 1669.04) | Male | 40.06 (-1.02 to 81.15) | Female | 46.71 (-4.5 to 97.91) | Male | 20.67 (3.94 to 37.4) | Female | 16.09 (4.03 to 28.15) |
| 2035 | Male | 678.75 (-110.65 to 1468.15) | Female | 794.25 (0 to 1769.56) | Male | 40.07 (-5.63 to 85.76) | Female | 46.72 (-10.25 to 103.68) | Male | 20.11 (2.06 to 38.16) | Female | 15.53 (2.63 to 28.43) |
| 2036 | Male | 679.42 (-192.91 to 1551.75) | Female | 795.12 (0 to 1873.37) | Male | 40.06 (-10.39 to 90.51) | Female | 46.71 (-16.2 to 109.62) | Male | 19.56 (0.22 to 38.9) | Female | 14.98 (1.27 to 28.69) |
| 2037 | Male | 680 (-278 to 1638) | Female | 795.82 (0 to 1980.37) | Male | 40.05 (-15.31 to 95.4) | Female | 46.69 (-22.34 to 115.72) | Male | 19.03 (-1.58 to 39.64) | Female | 14.45 (-0.03 to 28.93) |
| 2038 | Male | 680.7 (-365.94 to 1727.35) | Female | 796.71 (0 to 2091.33) | Male | 40.04 (-20.38 to 100.46) | Female | 46.68 (-28.68 to 122.05) | Male | 18.51 (-3.33 to 40.35) | Female | 13.94 (-1.27 to 29.15) |
| 2039 | Male | 681.58 (-456.73 to 1819.9) | Female | 797.85 (-0.01 to 2206.39) | Male | 40.05 (-25.61 to 105.7) | Female | 46.69 (-35.21 to 128.6) | Male | 18.01 (-5.03 to 41.05) | Female | 13.44 (-2.47 to 29.35) |
| 2040 | Male | 682.56 (-550.31 to 1915.42) | Female | 799.11 (-0.01 to 2325.21) | Male | 40.06 (-30.98 to 111.1) | Female | 46.71 (-41.93 to 135.36) | Male | 17.52 (-6.69 to 41.73) | Female | 12.96 (-3.6 to 29.53) |
| 2041 | Male | 683.43 (-646.5 to 2013.37) | Female | 800.23 (-0.01 to 2446.97) | Male | 40.07 (-36.49 to 116.63) | Female | 46.72 (-48.82 to 142.26) | Male | 17.04 (-8.3 to 42.37) | Female | 12.5 (-4.69 to 29.69) |
| 2042 | Male | 684.23 (-745.25 to 2113.71) | Female | 801.2 (-0.01 to 2571.62) | Male | 40.06 (-42.14 to 122.27) | Female | 46.72 (-55.88 to 149.31) | Male | 16.57 (-9.86 to 43) | Female | 12.05 (-5.72 to 29.82) |
| 2043 | Male | 685.15 (-846.72 to 2217.02) | Female | 802.36 (-0.01 to 2700.09) | Male | 40.07 (-47.93 to 128.08) | Female | 46.73 (-63.12 to 156.57) | Male | 16.11 (-11.36 to 43.59) | Female | 11.62 (-6.69 to 29.93) |
| 2044 | Male | 686.23 (-950.98 to 2323.44) | Female | 803.75 (-0.01 to 2832.57) | Male | 40.09 (-53.87 to 134.06) | Female | 46.75 (-70.55 to 164.05) | Male | 15.67 (-12.82 to 44.16) | Female | 11.2 (-7.61 to 30.01) |
| 2045 | Male | 687.4 (-1057.95 to 2432.75) | Female | 805.26 (-0.01 to 2968.74) | Male | 40.11 (-59.96 to 140.19) | Female | 46.78 (-78.16 to 171.73) | Male | 15.24 (-14.22 to 44.69) | Female | 10.79 (-8.48 to 30.07) |
| 2046 | Male | 688.52 (-1167.4 to 2544.43) | Female | 806.67 (-0.01 to 3107.76) | Male | 40.13 (-66.17 to 146.44) | Female | 46.81 (-85.93 to 179.54) | Male | 14.81 (-15.57 to 45.19) | Female | 10.4 (-9.3 to 30.1) |
| 2047 | Male | 689.59 (-1279.31 to 2658.5) | Female | 807.98 (-0.02 to 3249.63) | Male | 40.15 (-72.51 to 152.81) | Female | 46.82 (-93.84 to 187.49) | Male | 14.4 (-16.86 to 45.67) | Female | 10.02 (-10.07 to 30.11) |
| 2048 | Male | 690.79 (-1393.97 to 2775.56) | Female | 809.49 (-0.02 to 3395.38) | Male | 40.17 (-78.99 to 159.33) | Female | 46.85 (-101.95 to 195.66) | Male | 14 (-18.1 to 46.11) | Female | 9.65 (-10.79 to 30.09) |
| 2049 | Male | 692.15 (-1511.47 to 2895.76) | Female | 811.21 (-0.02 to 3545.25) | Male | 40.21 (-85.62 to 166.03) | Female | 46.9 (-110.25 to 204.04) | Male | 13.61 (-19.29 to 46.52) | Female | 9.3 (-11.45 to 30.05) |
| 2050 | Male | 693.58 (-1631.69 to 3018.86) | Female | 813.04 (-0.02 to 3698.83) | Male | 40.25 (-92.39 to 172.88) | Female | 46.95 (-118.73 to 212.62) | Male | 13.23 (-20.43 to 46.9) | Female | 8.96 (-12.08 to 29.99) |
| 2051 | Male | 694.97 (-1754.34 to 3144.29) | Female | 814.79 (-0.02 to 3855.22) | Male | 40.28 (-99.28 to 179.85) | Female | 46.99 (-127.35 to 221.33) | Male | 12.86 (-21.52 to 47.25) | Female | 8.62 (-12.65 to 29.9) |

Abbreviations: ASR, age-standardized rate; DALYs, disability-adjusted life-years; CrI, credible interval.
